# The impact of childhood injury and injury severity on school performance and high school completion in Australia: a matched population-based retrospective cohort study

**DOI:** 10.1186/s12887-021-02891-x

**Published:** 2021-09-25

**Authors:** Rebecca J. Mitchell, Cate M. Cameron, Anne McMaugh, Reidar P. Lystad, Tim Badgery-Parker, Tayhla Ryder

**Affiliations:** 1 Australian Institute of Health Innovation, Faculty of Medicine, Health and Human Sciences, Macquarie University, Level 6, 75 Talavera Road, Sydney, NSW 2109 Australia; 2 Jamieson Trauma Institute, Royal Brisbane & Women’s Hospital, Metro North Hospital and Health Services District, Brisbane, Australia; 3 Queensland University of Technology (QUT), Centre for Healthcare Transformation, Australian Centre for Health Services Innovation (AusHSI), Brisbane, Australia; 4 The Macquarie School of Education, Macquarie University, Sydney, Australia

**Keywords:** Injury, Academic performance, School completion

## Abstract

**Background:**

Exploring the impact of injury and injury severity on academic outcomes could assist to identify characteristics of young people likely to require learning support services. This study aims to compare scholastic performance and high school completion of young people hospitalised for an injury compared to young people not hospitalised for an injury by injury severity; and to examine factors influencing scholastic performance and school completion.

**Method:**

A population-based matched case-comparison cohort study of young people aged ≤18 years hospitalised for an injury during 2005–2018 in New South Wales, Australia using linked birth, health, education and mortality records. The comparison cohort was matched on age, gender and residential postcode. Generalised linear mixed modelling examined risk of performance below the national minimum standard (NMS) on the National Assessment Plan for Literacy and Numeracy (NAPLAN) and generalised linear regression examined risk of not completing high school for injured young people compared to matched peers.

**Results:**

Injured young people had a higher risk of not achieving the NMS compared to their matched peers for numeracy (ARR: 1.12; 95%CI 1.06–1.17), reading (ARR: 1.09; 95%CI 1.04–1.13), spelling (ARR: 1.13; 95%CI 1.09–1.18), grammar (ARR: 1.11; 95%CI 1.06–1.15), and writing (ARR: 1.07; 95%CI 1.04–1.11). As injury severity increased from minor to serious, the risk of not achieving the NMS generally increased for injured young people compared to matched peers. Injured young people had almost twice the risk of not completing high school at year 10 (ARR: 2.17; 95%CI 1.73–2.72), year 11 (ARR: 1.95; 95%CI 1.78–2.14) or year 12 (ARR: 1.93; 95%CI 1.78–2.08) compared to matched peers.

**Conclusions:**

The identification of characteristics of young people most likely to encounter problems in the academic environment after sustaining an injury is important to facilitate the potential need for learning support. Assessing learning needs and monitoring return-to-school progress post-injury may aid identification of any ongoing learning support requirements.

**Supplementary Information:**

The online version contains supplementary material available at 10.1186/s12887-021-02891-x.

## Background

Injury is one of the most common reasons for hospitalisation of young people, with millions worldwide sustaining a traumatic injury requiring hospitalisation each year [1, 2]. In Australia, around 70,000 young people aged ≤16 years are hospitalised annually following an injury [3]. An injury can have an adverse impact on a young person’s health, development, and school performance [4, 5]. The more serious the injury, the greater the negative impact on a young person’s psychological and physical health [6, 7]. Injury severity has also been found to influence post-injury academic progress [8], with traumatic brain injury (TBI) one of the most serious injuries experienced by a young person [9].

After sustaining an injury, a young person’s ability to learn and concentrate can be interrupted, with physical disability shown to negatively influence cognitive skills [10, 11]. Injured young people have been shown to have lower scores for academic achievement, especially following a TBI [5, 12]. Future learning can also be affected and problems with new skill acquisition can persist or worsen [13, 14]. Along with problems with cognitive performance, a seriously injured young person may also experience psychological and physical health problems that could adversely affect their academic performance [6, 7]. Education is critically important for a young person’s psychological, social, and physical development [15]. Poor academic performance at school can adversely affect both long-term career prospects and quality of life [16, 17]. Interrupted education can also have a cumulative effect, resulting in a young person not completing high school or undertaking tertiary studies, and therefore limiting future employment opportunities [14, 18].

Much of the research on academic outcomes of injured young people has focused on TBI, where young people with an orthopaedic injury are typically used as a control group to compare academic achievement [10, 11, 13, 19]. Comparing the academic performance of young people who sustained a TBI to those who had an orthopaedic injury assumes that orthopaedic injuries, and other injuries, do not have an adverse effect on school performance. It appears that orthopaedic injuries can have a negative effect on academic performance compared to healthy non-injured controls, but this effect is not as severe as the effect on young people who sustained a moderate or serious TBI [20].

No previous studies have examined the impact of all types of injury and injury severity on school performance and high school completion. Gaining an understanding of the association of injury and the severity of injury on academic outcomes could assist to identify characteristics of young people likely to require future educational support measures and guide the need for early interventions. This study aims to compare scholastic performance and high school completion of young people hospitalised for an injury compared to young people not hospitalised for an injury by injury severity; and to examine factors influencing scholastic performance and school completion.

## Method

A population-based case-comparison matched retrospective cohort study of young people injured and hospitalised aged ≤18 years in New South Wales (NSW), Australia using linked birth, health, education and mortality administrative data collections between 1 January 2005 and 31 December 2018 [21].

### Data sources

Information on hospital service use was obtained from emergency department (ED) and hospital admission data collections. ED visits to public hospitals included information on arrival and departure times, visit type, and provisional diagnosis. Data on hospital admissions included admissions to both public and private hospitals, child demographics, diagnoses, separation type (i.e. statistical discharge, death), and clinical procedures. Mortality data was obtained for the study time period from the NSW Registry of Births, Deaths and Marriages and young people who died during the study period were excluded from analyses.

School performance data and parental demographics were obtained from the annual National Assessment Plan for Literacy and Numeracy (NAPLAN) assessments conducted in May from 2008 to 2018 for government, Catholic, and independent schools [22]. Assessments were conducted on all young people in primary school grades 3 (7–9 years of age) and 5 (9–11 years of age), and secondary school grades 7 (11–13 years of age) and 9 (13–15 years of age), and include assessments of learning in five domains: numeracy, reading, spelling, writing, and grammar and punctuation (Supplementary Fig. 1). Each domain is scored out of 1000 and assessment scores represent the same level of achievement over time [23]. Each score is translated into proficiency bands that indicate whether the child performed above, at, or below the national minimum standard (NMS). Inability to achieve the NMS indicates that a child will have difficulty making progress in school without assistance [24].

Information on a child’s attendance, absence, withdrawal (e.g. philosophical objections to testing or religious beliefs) or exemption due to significant disability that prevented the child from completing an assessment was obtained (Supplementary Table 1). Young people who were exempt from sitting a NAPLAN assessment due to severe disability or language difficulties were rated as scoring below NMS in accordance with technical guidelines [25]. From 2011, the writing assessment task changed from a narrative to a persuasive assessment task, so pre- and post-2011 writing assessment results were not comparable [26]. Only the writing persuasive assessment was examined for this study.

A young person was identified as having a language background other than English (LBOTE) if either they or their parents/guardians spoke a language other than English at home [23]. Where there were multiple records of the parents’ level of education, the highest level of education of either parent was identified. Information on high school completions at years 10 (15–16 years of age), 11 (17–18 years of age) and 12 (17–18 years of age) were obtained through the Record of School Achievement, and the Higher School Certificate (Supplementary Fig. 2).

The Centre for Health Record Linkage (CHeReL) identified the population comparison group and linked the health and education records using probabilistic record linkage. Upper and lower probability cut-offs for a link were 0.75 and 0.25 and record groups with probabilities between the cut-offs were clerically reviewed.

### Case inclusion criteria

The injured cohort included young people with a year of birth ≥1997 who were aged ≤18 years at hospital admission with a principal diagnosis of injury (International Classification of Diseases, 10th Revision, Australian Modification (ICD-10-AM): S00-T75 or T79) between 1 January 2005 and 31 December 2018. Cases were included if their hospitalised injury occurred before their NAPLAN assessment date (allocated to 15 May of each year) (i.e. the young person was injured and hospitalised prior to their NAPLAN assessment) and the young person completed all five NAPLAN domain assessments in the examination period. For school completions, cases were included if the hospitalised injury occurred before the school completion date (allocated to 19 December). The ICD-10-AM external cause codes (i.e. V00-Y36) were used to describe the mechanism of injury.

### Population-comparison group criteria

The comparison cohort included young people who were not hospitalised for an injury between 1 July 2001 and 31 December 2018. They were randomly selected from NSW birth records and matched 1:1 on age, gender and residential postcode to their injured counterpart. The comparison selection timeframe included a 3.5 year wash-out period prior to the case selection timeframe to avoid potential selection of comparisons who had been hospitalised for an injury prior to the case criteria timeframe.

### Geographical location and socioeconomic status

The Australian Statistical Geographical Standard was used to assign the young person’s residential postcode to one of five geographical categories using index scores of distance to service centres [27]. The five categories were classified as urban (i.e. major cities) and rural (i.e. inner and outer regional, remote, and very remote). The remoteness area of school was obtained from NAPLAN records and was categorised as major city, inner regional, outer regional/remote [27]. Socioeconomic status was assigned with the Index of Relative Socioeconomic Disadvantage [28] using the young person’s postcode of residence; socioeconomic disadvantage was partitioned into quintiles from most (i.e. 1) to least disadvantaged (i.e. 5). In the majority of cases (89.3%), the young person’s postcode of residence was assigned using the postcode of residence at birth. Where this was not possible, the remaining postcodes (10.7%) were assigned using the young person’s hospital or other health records.

### Injury severity and chronic health conditions

The International Classification of Injury Severity Score (ICISS) [29] was used to estimate injury severity. The injury severity score is derived for each injured young person by multiplying the probability of survival for each injury diagnosis in the hospitalisation records using survival risk ratios. Injury severity was estimated using previously developed survival risk ratios [30] and was categorised as minor (≥0.99), moderate (> 0.941- < 0.99) or serious (≤0.941) [31]. A TBI was identified using a principal ICD-10-AM diagnosis code of S06 in hospitalisation records.

Health conditions common for young people were identified from the literature [32–34]. A chronic condition would reasonably be expected to last 12 months or result in the need for ongoing health care [32]. For this study, a chronic condition was identified using diagnosis classifications from ICD-10-AM (Supplementary Table 2) in the hospitalisation records. A 3-year look-back period to 1 January 2002 was used for the identification of chronic health conditions.

### Data organisation and analysis

Data analysis was conducted using SAS 9.4 (SAS Institute, Cary NC) and RStudio V1.2.5001 (RStudio Inc). All hospital episodes of care related to the one event (e.g. all episodes of care related to the same injury event) were linked to form a period of health care. Chi-square tests of independence and Wilcoxon Mann-Whitney tests were used to examine characteristics of injured young people and their non-injured counterparts. The number of ED visits, hospital admissions and hospital length of stay (LOS) during and after the index injury admission were identified for both the injured young person and their non-injured counterpart before each NAPLAN assessment. The calculation of hospital LOS was cumulative and included transfers between hospitals. The index admission was included in the counts of ED visits, hospitalisations and cumulative hospital LOS.

To examine NAPLAN assessment performance at each grade, generalised linear regression using PROC GENMOD assessed the difference in proportions of performances below the NMS for each of the five NAPLAN domains for the school grades 3, 5, 7 and 9 for injured young people and their matched counterparts (Supplementary Tables 3 and 4). For each domain, models were fitted using generalised estimating equations (GEE) with binomial distribution, and a log function. Adjusted relative risks (ARR) and 95% confidence intervals (CIs) were calculated. For each domain, forward selection was used to sequentially add covariates to the model and significance was assessed using *p*-values (*p* < 0.05) to examine the overall effect in the model. The a priori model included age, gender, socioeconomic status of residential area, geographic location, chronic health condition, parental highest level of education, parental occupation, and number of ED visits or number of hospitalisations or total hospital LOS [21]. The final models included injury status, gender, comorbidity status (Y/N), LBOTE, socioeconomic status of residential area, highest level of education for any parent/guardian (i.e. bachelor or higher degree or other), and a log of hospital LOS. As comparison group members could have nil hospital LOS, a constant value was added to LOS before transformation [35].

Generalised linear mixed modelling (GLMM) was conducted to perform multi-level modelling of NAPLAN performance below the NMS for each of five NAPLAN domains for young people and their counterpart who had completed multiple grades of schooling. For each domain, PROC GLIMMIX was used with a binary distribution, log link function, and Kenward and Roger denominator degrees of freedom. The residual option of the random statement was used to model R-side covariance and data were analysed to account for within student correlation in the longitudinal data and repeated measurements using an autoregressive covariance structure. ARRs and 95% CIs were generated. The final model included: injury status, NAPLAN grade (i.e. 3, 5, 7 or 9), gender, comorbidity status (Y/N), LBOTE, socioeconomic status of residential area, highest level of education for any parent/guardian (i.e. bachelor or higher degree or other), log of hospital LOS, and school sector (i.e. government, Catholic, independent).

Factors associated with high school completion at either year 10, 11 or 12 for injured young people compared to their counterparts were examined using generalised linear regression using PROC GENMOD. For each grade, models were fitted using GEE with binomial distribution, and a log function. ARR and 95% CIs were calculated. For each grade, forward selection was used to sequentially add covariates to the model and significance was assessed using *p*-values (*p* < 0.05) to examine the overall effect in the model. The final models included injury status, gender, comorbidity status (Y/N), LBOTE, socioeconomic status of residential area, highest level of education for any parent/guardian, and injury x comorbidity status interaction.

## Results

There were 50,213 young people hospitalised for an injury prior to completing their NAPLAN assessment in Grade 3, 46,034 in Grade 5, 36,962 in Grade 7, and 24,501 in Grade 9. There were 43,987 young people who had an injury hospitalisation with a non-injured matched comparison who could have completed year 10; 41,454 year 11; and 34,255 year 12 of high school.

Prior to their Grade 5 to 9 NAPLAN assessments, there was a higher proportion of injury hospitalisations of males (56.7–60.9%) and of young people residing in urban locations (71.7–73.3%). All injured young people had a higher proportion of experiencing ≥1 health condition and health care use compared to their matched peers. However, the proportion of comorbidities identified was generally low (i.e. < 1%). A higher proportion of injured young people prior to their Grades 3 and 5 NAPLAN assessments were non-LBOTE compared to their matched non-injured comparison. Injured young people prior to their Grade 3 to 7 NAPLAN assessments had a lower proportion of parents with a tertiary degree as their highest level of education compared to their matched peers (Table 1).

**Table 1:** Demographic and healthcare use characteristics of injured young people and their matched comparison by grade, linked health and school performance data NSW, 2005–2018

	Grade 3^1^				Grade 5^2^				Grade 7^3^				Grade 9^4^			
Characteristics	Injury case (*n* = 50,213)		Comparison (*n* = 50,213)		Injury case (*n* = 46,034)		Comparison (*n* = 46,034)		Injury case (*n* = 36,962)		Comparison (*n =* 36,962)		Injury case (*n* = 24,501)		Comparison (*n* = 24,501)	
	n	%	n	%	n	%	n	%	n	%	n	%	n	%	n	%
**Gender**																
Male	28,473	56.7	28,473	56.7	26,221	57.0	26,221	57.0	21,392	57.9	21,392	57.9	14,924	60.9	14,924	60.9
Female	21,740	43.3	21,740	43.3	19,813	43.0	19,813	43.0	15,570	42.1	15,570	42.1	9577	39.1	9577	39.1
**Location of residence**																
Urban	36,295	72.3	36,295	72.3	32,986	71.7	32,986	71.7	26,736	72.3	26,736	72.3	17,966	73.3	17,966	73.3
Rural	13,893	27.7	13,893	27.7	13,027	28.3	13,027	28.3	10,209	27.6	10,209	27.6	6517	26.6	6517	26.6
Not known	25	0.1	25	0.1	21	0.1	21	0.1	17	0.1	17	0.1	18	0.1	18	0.1
**Socioeconomic status**																
Most disadvantaged	10,658	21.2	10,658	21.2	9581	20.8	9581	20.8	7507	20.3	7507	20.3	4737	19.3	4737	19.3
2	11,450	22.8	11,450	22.8	10,568	23.0	10,568	23.0	8324	22.5	8324	22.5	5315	21.7	5315	21.7
3	10,890	21.7	10,890	21.7	10,118	22.0	10,118	22.0	8151	22.1	8151	22.1	5400	22.0	5400	22.0
4	5306	10.6	5306	10.6	4782	10.4	4782	10.4	3933	10.6	3933	10.6	2706	11.0	2706	11.0
Least disadvantaged	11,882	23.7	11,882	23.7	10,962	23.8	10,962	23.8	9029	24.4	9029	24.4	6324	25.8	6324	25.8
Not known	27	0.1	27	0.1	24	0.1	24	0.1	18	0.1	18	0.1	19	0.1	19	0.1
**LBOTE**^5^																
Non-LBOTE	39,072	77.8	38,669	77.0	36,710	79.8	36,365	79.0	29,850	80.8	29,629	80.2	19,815	80.9	19,695	80.4
LBOTE	10,889	21.7	11,299	22.5	9213	20.0	9569	27.8	7067	19.1	7280	19.7	4662	19.0	4778	19.5
Not known	252	0.5	245	0.5	111	0.2	100	0.2	45	0.1	53	0.1	24	0.1	28	0.1
**Health condition**																
0	49,875	99.3	49,577	98.7	45,707	99.3	45,382	95.6	36,367	99.3	36,393	98.5	24,320	99.3	24,042	98.1
1	323	0.6	624	1.2	311	0.7	644	1.4	255	0.7	560	1.5	174	0.7	454	1.9
≥2	15	0.03	12	0.02	16	0.03	8	0.02	10	0.03	9	0.02	7	0.03	5	0.02
**Parent highest level of education**																
Year 11 or equivalent	3714	7.4	3226	6.4	4230	9.2	3685	8.0	3139	8.5	2817	7.6	2003	8.2	1868	7.6
Year 12 or equivalent	2042	4.1	1950	3.9	2315	5.0	2241	4.9	1796	4.9	1743	4.7	1211	4.9	1221	5.0
Certificate I-IV or trade	14,716	29.3	14,596	29.1	13,704	29.8	13,723	29.8	11,122	30.1	11,172	30.2	7319	29.9	7315	18.3
Advanced diploma/ diploma	8317	16.6	8438	16.8	7846	17.0	17,795	38.7	6625	17.9	6576	17.8	9205	37.6	9301	38.0
Bachelor degree or higher	18,880	37.6	19,717	39.3	17,260	34.5	17,795	38.7	13,856	37.5	14,250	38.6	9205	37.6	9301	38.0
Not stated/not known	2544	5.1	2286	4.6	679	1.5	630	1.4	424	1.2	404	1.1	296	1.2	320	1.3
**Health care use**	**Mean**	**SD**	**Mean**	**SD**	**Mean**	**SD**	**Mean**	**SD**	**Mean**	**SD**	**Mean**	**SD**	**Mean**	**SD**	**Mean**	**SD**
ED visits	6.7	6.1	3.7	3.0	6.8	6.2	4.6	4.4	6.6	6.0	4.5	4.4	6.2	5.7	4.1	3.9
Hospital admissions	2.8	4.2	1.1	3.2	2.8	4.6	2.0	3.7	2.6	3.7	2.0	2.8	2.4	3.9	1.8	2.9
Hospital length of stay	6.3	19.3	3.4	9.7	6.1	20.6	3.3	10.5	5.4	16.0	2.9	10.6	4.5	14.7	2.2	7.3

^1^ Grade 3 chi-square tests: LBOTE *p* < 0.008; Health conditions *p* < 0.0001; Parent highest level of education *p* < 0.0001; and Wilcoxon Mann-Whitney tests: ED visits *p* < 0.0001; Hospital admissions *p* < 0.0001; and Hospital length of stay *p* < 0.0001. ^2^ Grade 5 chi-square tests: LBOTE *p* < 0.01; Health conditions *p* < 0.0001; Parent highest level of education *p* < 0.0001; and Wilcoxon Mann-Whitney tests: ED visits *p* < 0.0001; Hospital admissions *p* < 0.0001; and Hospital length of stay *p* < 0.0001. ^3^ Grade 7 chi-square tests: LBOTE *p* = 0.1; Health conditions *p* < 0.0001; Parent highest level of education *p* < 0.0002; and Wilcoxon Mann-Whitney tests: ED visits *p* < 0.0001; Hospital admissions *p* < 0.0001; and Hospital length of stay *p* < 0.0001. ^4^ Grade 9 chi-square tests: LBOTE *p* = 0.4; Health conditions p < 0.0001; Parent highest level of education *p* = 0.3; and Wilcoxon Mann-Whitney tests: ED visits *p* < 0.0001; Hospital admissions *p* < 0.0001; and Hospital length of stay *p* < 0.0001. ^5^Language background other than English

Compared to their matched peers, the school sector profile and remoteness area of the school differed for injured young people, excluding school sector for Grade 9. However, both injured and non-injured young people predominantly attended government schools in major cities. A higher proportion of injured young people did not achieve the NMS for their NAPLAN assessments in Grades 3 to 9 compared to their non-injured counterpart (Table 2). Falls, minor injuries, injuries to the head or elbow and forearm, and fractures or open wounds were the most common injury characteristics for injured young people. Minor injuries accounted for around 95%, moderate injuries around 4% and serious injuries around 1% of hospital admissions of injured young people. Months since the index injury hospitalisation for the injured young people ranged from a mean of 46.2 prior to their Grade 3 NAPLAN assessment to a mean of 70.0 for their Grade 9 NAPLAN assessment (Table 3).

**Table 2:** School and NAPLAN assessment characteristics of injured young people and their matched comparison by grade, linked health and school performance data NSW, 2005–2018

	Grade 3^1^				Grade 5^2^				Grade 7^3^				Grade 9^4^			
Characteristics	Injury case (*n* = 50,213)		Comparison (*n* = 50,213)		Injury case (*n* = 46,034)		Comparison (*n* = 46,034)		Injury case (*n* = 36,962)		Comparison (*n* = 36,962)		Injury case (*n* = 24,501)		Comparison (*n* = 24,501)	
	n	%	n	%	n	%	n	%	n	%	n	%	n	%	n	%
**School sector**																
Government	35,269	70.2	34,741	69.2	31,678	68.8	30,979	67.3	21,133	57.2	20,872	56.5	13,685	55.9	13,660	55.8
Catholic	9774	19.5	10,455	20.8	8986	19.5	9806	21.3	9752	26.4	10,108	27.4	6778	27.7	6842	27.9
Independent	5153	10.3	5000	10.0	5353	11.6	5237	11.4	6065	16.4	5974	16.2	4033	16.5	3995	16.3
Home	17	0.03	17	0.03	17	0.04	12	0.03	12	0.03	8	0.02	5	0.02	4	0.02
**Remoteness area of school**																
Major city	35,463	70.6	35,000	69.7	32,256	70.1	31,753	69.0	26,838	72.6	26,318	71.2	18,077	73.8	17,709	72.3
Inner regional	10,993	21.9	11,506	22.9	10,314	22.4	10,863	23.6	7890	21.4	8453	22.9	5108	20.9	5500	22.5
Outer regional/remote	3740	7.5	3690	7.4	3447	7.5	3406	7.4	2222	6.0	2183	5.9	1311	5.4	1288	5.3
Not known	17	0.03	17	0.03	17	0.04	12	0.03	12	0.03	8	0.02	5	0.02	4	0.02
**NAPLAN assessment**^5^																
Numeracy (Below NMS)	2734	5.4	1966	3.9	2786	6.1	1981	4.3	1609	4.4	1086	2.9	811	3.3	610	2.5
Reading (Below NMS)	3208	6.4	2283	4.6	3584	7.8	2647	5.8	2225	6.0	1723	4.7	1870	7.6	1454	5.9
Spelling (Below NMS)	3191	6.4	2253	4.5	3460	7.5	2473	5.4	2899	7.8	2203	6.0	2490	10.2	1916	7.8
Grammar (Below NMS)	3622	7.2	2595	5.2	3940	8.6	2879	6.3	3404	9.2	2569	7.0	2749	11.2	2198	9.0
Writing (Persuasive 2011–2018) (Below NMS)	1851	4.3	1251	2.9	4184	9.6	3014	6.9	4412	11.9	3473	9.4	4349	17.8	3695	15.1

^1^ Grade 3 chi-square tests: School sector *p* < 0.0001; Remoteness area of school *p* < 0.002; All NAPLAN assessments *p* < 0.0001. ^2^ Grade 5 chi-square tests: School sector *p* < 0.02; Remoteness area of school *p* < 0.0002. All NAPLAN assessments *p* < 0.0001. ^3^ Grade 7 chi-square tests: School sector *p* < 0.0001; Remoteness area of school *p* < 0.0003. All NAPLAN assessments *p* < 0.0001. ^4^ Grade 9 chi-square tests: School sector *p* = 0.9; Remoteness area of school *p* < 0.002. All NAPLAN assessments *p* < 0.0001. ^5^ *NAPLAN* National Assessment Plan for Literacy and Numeracy; *NMS* National Minimum Standard.

**Table 3:** Injury characteristics of injured young people by grade, linked health and school performance data NSW, 2005–2018

Characteristic	Injury cohort – Grade 3 (*n* = 50,213)		Injury cohort – Grade 5 (*n* = 46,034)		Injury cohort – Grade 7 (*n* = 36,962)		Injury cohort – Grade 9 (*n* = 24,501)	
	n	%	n	%	n	%	n	%
**Injury mechanism**								
Transport incidents	3753	7.5	4302	9.4	4007	10.8	2985	12.2
Falls	24,891	49.6	22,962	49.9	18,544	50.2	12,069	49.3
*Fall from playground equipment*	*7223*	*14.4*	*6519*	*14.2*	*4932*	*13.3*	*2947*	*12.0*
Inanimate mechanical forces	11,051	22.0	9591	20.8	7309	19.8	4447	18.2
Animate mechanical forces	2274	4.5	2145	4.7	1817	4.9	1430	5.8
Drowning and submersion or other threats to breathing	523	1.0	376	0.8	237	0.6	95	0.4
Smoke, fire, flames, heat and hot substances	1937	3.9	1494	3.3	949	2.6	424	1.7
Venomous animals and plants	369	0.7	373	0.8	281	0.8	190	0.8
Poisoning	1970	3.9	1541	3.4	968	2.6	374	1.5
Assault	283	0.6	239	0.5	166	0.5	125	0.5
Other and unspecified injury mechanism	3162	6.3	3011	6.5	2684	7.3	2362	9.6
**Injury severity**								
Minor (ICISS ≤0.99)	47,577	94.8	43,795	95.1	35,201	95.2	23,364	95.4
Moderate (ICISS 0.942–0.99)	1925	3.8	1668	3.6	1332	3.6	860	3.5
Serious (ICISS < 0.942)	711	1.4	571	1.2	429	1.2	277	1.1
**Traumatic brain injury** (yes)	1337	2.7	1268	2.8	1117	3.0	809	3.3
**Principal injury type**								
Head	13,606	27.1	11,115	24.2	7916	21.4	4637	18.9
Neck	452	0.9	548	1.2	554	1.5	466	1.9
Thorax	170	0.3	193	0.4	199	0.5	163	0.7
Abdomen, lower back, lumbar spine and pelvis	992	2.0	1048	2.3	917	2.5	664	2.7
Shoulder and upper arm	5342	10.6	4615	10.0	3393	9.2	2165	8.8
Elbow and forearm	11,049	22.0	11,934	25.9	10,757	29.1	7573	30.9
Wrist and hand	5004	10.0	4737	10.3	4045	10.9	2920	11.9
Hip and thigh	1148	2.3	991	2.2	824	2.2	517	2.1
Knee and lower leg	2131	4.2	2338	5.1	2388	6.5	2162	8.8
Ankle and foot	2087	4.2	1998	4.3	1656	4.5	1091	4.5
Other injuries^a^	8232	16.4	6517	14.2	4313	11.7	2143	8.8
**Nature of principal injury**								
Superficial injuries	3052	6.1	2629	5.7	1888	5.1	1140	4.7
Open wound	10,801	21.5	9270	20.1	6848	18.5	4008	16.4
Fracture	21,220	42.3	21,196	46.0	18,580	50.3	13,397	54.7
Dislocations, sprains and strains	543	1.1	580	1.3	532	1.4	533	2.2
Injury to nerves and spinal cord	225	0.5	213	0.5	179	0.5	140	0.6
Injury of eye and orbit	258	0.5	246	0.5	204	0.6	123	0.5
Injury to blood vessels	64	0.1	63	0.1	62	0.2	41	0.2
Injury to muscle, fascia and tendon	462	0.9	492	1.1	441	1.2	344	1.4
Crushing injury	241	0.5	191	0.4	133	0.4	79	0.3
Traumatic amputation	538	1.1	426	1.1	318	0.9	165	0.7
Injury to internal organs	1502	3.0	1469	3.2	1303	3.5	957	3.9
Foreign body entering through natural orifice	3058	6.1	2376	5.2	1576	4.3	808	3.3
Burns	2148	4.3	1661	3.6	1077	2.9	476	1.9
Poisoning by drugs, medicaments and biological substances	1574	3.1	1242	2.7	785	2.1	371	1.5
Toxic effects of substances chiefly nonmedicinal as to source	738	1.5	644	1.4	444	1.2	248	1.0
Other and unspecified injuries	3789	7.6	3336	7.3	2592	7.0	1671	6.8
	**mean**	**SD**	**mean**	**SD**	**mean**	**SD**	**mean**	**SD**
**Time since index injury hospitalisation to NAPLAN assessment** (months)	46.2	27.4	57.8	33.3	66.3	39.4	70.0	42.9

^a^Other injuries include: injuries involving multiple body regions, injuries to unspecified parts of trunk, limb or body region, effects of foreign bodies, burns, frostbite, poisoning, and unspecified injuries.

For each Grade, generalised linear regression indicated that injured young people had a higher risk of obtaining a NAPLAN assessment result below the NMS in each of the five NAPLAN domains compared to their matched peers. Generally, for young people who sustained a TBI, and as injury severity increased, the risk of not reaching the NMS increased for NAPLAN assessments in Grades 3 and 5 compared to their matched peers (Supplementary Table 3). The relationship of injury severity was less clear for injured young people for NAPLAN assessments in Grades 7 and 9 (Supplementary Table 4).

For all injuries, multi-level modelling indicated the risk of not achieving the NMS was higher for injured young people compared to their matched peers for the numeracy (ARR: 1.12; 95%CI 1.06–1.17), reading (ARR: 1.09; 95%CI 1.04–1.13), spelling (ARR: 1.13; 95%CI 1.09–1.18), grammar (ARR: 1.11; 95%CI 1.06–1.15), and writing (ARR: 1.07; 95%CI 1.04–1.11) NAPLAN assessments (Table 4). For young people who had multiple injury hospitalisations during the study time period, the ARRs were higher than those for all injuries as the risk of not achieving the NMS for NAPLAN assessments was higher for numeracy (ARR: 1.27; 95%CI 1.14–1.43), reading (ARR: 1.27; 95%CI 1.16–1.40), spelling (ARR: 1.29; 95%CI 1.17–1.41), grammar (ARR: 1.22; 95%CI 1.12–1.33), and writing (ARR: 1.28; 95%CI 1.18–1.39) compared to their matched counterparts.

**Table 4:** Multilevel model of characteristics associated with a below NMS NAPLAN assessment for young people with an index injury hospitalisations during 2005–2018 compared to a matched comparison by assessment, linked health and school performance data NSW

	Numeracy^1^		Reading^1^		Spelling^1^		Grammar^1^		Writing (Persuasive 2011–2018) ^1^	
	ARR^2^	95%CI	ARR^2^	95%CI	ARR^2^	95%CI	ARR^2^	95%CI	ARR^2,3^	95%CI
**Injury**										
No	1		1		1		1		1	
Yes	1.12	1.06–1.17	1.09	1.04–1.13	1.13	1.09–1.18	1.11	1.06–1.15	1.07	1.04–1.11
**Gender**										
Male	1		1		1		1		1	
Female	0.80	0.77–0.82	0.55	0.53–0.57	0.49	0.47–0.50	0.56	0.54–0.57	0.41	0.40–0.42
**Health condition**										
No	1		1		1		1			
Yes	1.78	1.58–2.00	1.60	1.45–1.78	1.59	1.44–1.75	1.53	1.39–1.68	–	–
**LBOTE**										
No	1		1		1		1		1	
Yes	0.91	0.87–0.95	0.83	0.80–0.86	0.62	0.59–0.64	0.81	0.78–0.83	0.63	0.61–0.65
**Socioeconomic status**										
Most disadvantaged	0.31	0.29–0.34	0.33	0.31–0.35	0.36	0.34–0.38	0.37	0.35–0.39	0.39	0.38–0.41
2	0.40	0.37–0.43	0.42	0.40–0.44	0.44	0.42–0.47	0.45	0.43–0.48	0.46	0.44–0.48
3	0.50	0.46–0.53	0.50	0.47–0.53	0.53	0.51–0.56	0.54	0.51–0.57	0.55	0.53–0.58
4	0.62	0.57–0.68	0.63	0.59–0.68	0.67	0.63–0.72	0.66	0.62–0.70	0.67	0.63–0.71
Least disadvantaged	1		1		1		1		1	
**NAPLAN Grade**										
3	1		1		1		1		1	
5	1.32	1.27–1.38	1.18	1.14–1.22	0.88	0.85–0.90	0.86	0.84–0.89	0.72	0.71–0.74
7	1.60	1.51–1.69	0.90	0.87–0.94	0.67	0.64–0.69	0.69	0.67–0.71	0.48	0.47–0.50
9	1.12	1.07–1.16	1.24	1.20–1.29	1.19	1.16–1.23	1.20	1.17–1.24	2.21	2.13–2.30
**Parental education**										
Bachelor/higher degree	1		1		1		1		1	
Other	0.37	0.35–0.39	0.35	0.33–0.36	0.40	0.38–0.42	0.37	0.36–0.39	0.42	0.41–0.44
**School sector**										
Government	1		1		1		1		1	
Catholic	1.02	0.92–1.12	0.98	0.91–1.06	1.01	0.94–1.07	1.03	0.97–1.10	0.92	0.87–0.97
Independent	0.41	0.39–0.44	0.46	0.44–0.48	0.54	0.52–0.56	0.50	0.49–0.52	0.47	0.45–0.49

^1^Numeracy, reading, spelling, grammar, and writing type III tests of fixed effects: Injury *p*<0.0001; Gender *p*<0.0001; Health condition *p*<0.0001; LBOTE *p*<0.0001; socioeconomic status *p*<0.0001; NAPLAN grade *p*<0.0001; Parental education *p*<0.0001; School sector *p*<0.0001; and hospital LOS p<0.0001. ^2^Adjusted relative risk excludes 176 with missing socioeconomic status, 858 with missing LBOTE and 92 home schooled. ^3^Writing excludes comorbidity status due to low cell size.

**Fig. 1:**
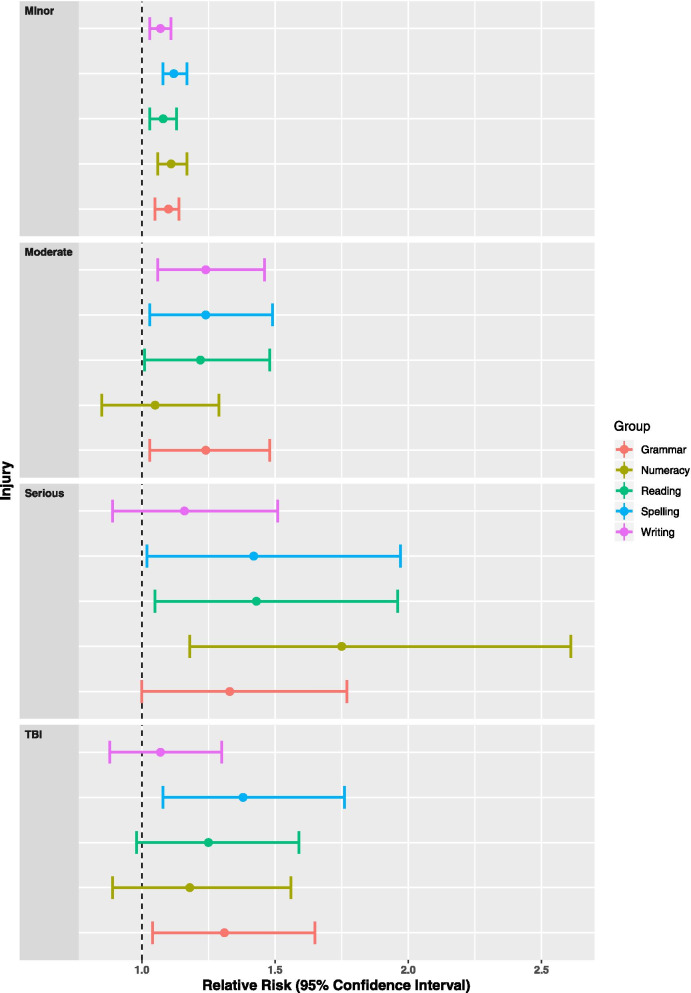
Multilevel model of characteristics associated with a below NMS NAPLAN assessment for young people by injury severity or with a TBI-related hospitalisation during 2005–2018 compared to a matched comparison by assessment, linked health and school performance data NSW ^1–4^ (^1^Minor injury adjusted relative risk excludes 174 with missing socioeconomic status, 808 with missing LBOTE and 90 home schooled and writing excludes comorbidity status due to low cell size. ^2^Moderate injury adjusted relative risk excludes 2 with missing socioeconomic status, 43 with missing LBOTE and 2 home schooled. Spelling excludes comorbidity status and writing (persuasive) excludes comorbidity status and LBOTE due to small cell sizes. ^3^Serious adjusted relative risk excludes 7 with missing LBOTE. Grammar excludes comorbidity status and writing (persuasive) excludes comorbidity status and LBOTE due to small cell sizes and school sector. ^4^Traumatic brain injury (TBI) adjusted relative risk excludes 10 with missing socioeconomic status, 19 with missing LBOTE and 4 home schooled. Grammar and writing (persuasive) exclude comorbidity status due to small cell sizes.)

Disaggregation by injury severity showed an increasing risk for injured young people of not achieving the NMS for NAPLAN assessments compared to their matched peers, except for the numeracy assessment for moderate injuries and the writing task for serious injuries (). Young people who sustained a TBI had an increased risk of not achieving the NMS for the spelling (ARR: 1.38; 95%CI 1.08–1.76) and grammar (ARR: 1.31; 95%CI 1.04–1.65) assessments compared to their matched peers (Fig. 1 and Supplementary Tables 5–8).

Young people who were hospitalised for an injury had a higher risk of not completing year 10 (ARR: 2.17; 95%CI 1.73–2.72), year 11 (ARR: 1.95; 95%CI 1.78–2.14) or year 12 (ARR: 1.93; 95%CI 1.78–2.08) compared to their matched peers (Table 5).

**Table 5:** Analysis of characteristics associated with not completing high school for young people with an index injury hospitalisations compared to a matched comparison by grade, linked health and school performance data NSW, 2005–2018

	Year 1^1^ *n* = 43,987 in each cohort		Year 11^2^ *n* = 41,451 in each cohort		Year 12^3^ *n* = 34,255 in each cohort	
	n	%^4^	n	%^4^	n	%^4^
**No school completion**						
Injured cohort	1723	3.9	9752	23.5	10,475	30.6
Non-injured cohort	991	2.3	5996	14.5	6274	18.3
	**Mean (median)**	**SD**	**Mean** **(median)**	**SD**	**Mean (median)**	**SD**
**Time since index injury hospitalisation to school completion** (months)	65.5 (60.4)	42.7	69.6 (65.0)	43.5	72.9 (69.4)	43.9
**Characteristics**	**ARR**^5^	**95%CI**	**ARR**^5^	**95%CI**	**ARR**^5^	**95%CI**
**Injury**						
No	1		1		1	
Yes	2.17	1.73–2.72	1.95	1.78–2.14	1.93	1.78–2.08
**Gender**						
Male	1		1		1	
Female	0.91	0.85–0.99	0.81	0.78–0.83	0.85	0.83–0.87
**Health condition**						
No	1		1		1	
Yes	1.63	1.30–2.04	1.19	1.09–1.31	1.26	1.17–1.36
**LBOTE**						
No	1		1		1	
Yes	0.76	0.54–1.06	0.87	0.76–0.99	0.82	0.71–0.92
Not known	0.59	0.50–0.69	0.66	0.62–0.69	0.69	0.65–0.72
**Socioeconomic status**						
Most disadvantaged	0.49	0.43–0.55	0.66	0.63–0.69	0.65	0.63–0.67
2	0.58	0.52–0.66	0.69	0.66–0.72	0.67	0.65–0.70
3	0.73	0.64–0.83	0.73	0.70–0.76	0.71	0.69–0.74
4	0.80	0.68–0.93	0.78	0.75–0.83	0.78	0.74–0.81
Least disadvantaged	1		1		1	
**Parental education**						
High school	0.80	0.58–1.10	0.75	0.66–0.86	0.69	0.61–0.79
Certificate I-IV, trade, diploma	1.73	1.26–2.36	0.90	0.79–1.03	0.82	0.73–0.93
Bachelor/higher degree	1		1		1	
Not stated	3.06	2.20–4.24	1.47	1.28–1.68	1.20	1.05–1.36

^1^Year 10 type III GEE analysis: Injury *p* < 0.0001; Gender *p* < 0.02; Health condition *p* < 0.0008; LBOTE *p* < 0.0001; socioeconomic status *p* < 0.0001; Parental education *p* < 0.0001; and Injury x health condition *p* < 0.05

^2^Year 11 type III GEE analysis: Injury *p* < 0.0001; Gender *p* < 0.0001; Health condition *p* < 0.0004; LBOTE *p* < 0.0001; socioeconomic status *p* < 0.0001; Parental education *p* < 0.0001; and Injury x health condition *p* < 0.0001

^3^Year 12 type III GEE analysis: Injury *p* < 0.0001; Gender *p* < 0.02; Health condition *p* < 0.0008; LBOTE *p* < 0.0001; socioeconomic status *p* < 0.0001; Parental education *p* < 0.0001; and Injury x health condition *p* < 0.0001

^4^ Percent calculated for young people in injury and comparison cohorts not completing the school grade.

^5^Adjusted relative risk excludes missing socioeconomic status

## Discussion

This research compared the scholastic performance and high school completion of injured young people and their non-injured matched peers and examined factors influencing scholastic performance and school completion. It identified that injured young people had a higher risk of not achieving the NMS in all five NAPLAN domains (i.e. numeracy, reading, spelling, grammar, and writing), that as injury severity increased the risk of not achieving the NMS generally increased, and for injured young people with a TBI the risk of not achieving the NMS for spelling and grammar increased, compared to their matched peers. Injured young people had almost twice the risk of not completing high school compared to their matched counterparts.

This study identified that a potential relationship exists between hospitalised injury, injury severity and academic performance, even after controlling for pre-injury factors, such as socioeconomic status, pre-existing health conditions, and parental education. This study suggests that, while some young people might fully recover after their injury, others may experience ongoing adverse effects in their school-based academic performance. This implies that early recognition of a young person’s need for learning support at school and early intervention could be critical to assist injured students perform at their best academically [11, 36].

Previous research has identified an association between orthopaedic [20] and burn [4] injuries and scholastic performance. Babikian et al. [20] examined the neurological performance of young people who had mild TBI compared to those with an orthopaedic injury and to non-injured young people, and found that young people with either a mild TBI or an orthopaedic injury performed similarly on neurological measures and that both injured groups performed worse than the non-injured group, suggestive of a general injury effect. While the effect of injury on academic performance is a multifactorial and complex association, it is possible that being hospitalised for an injury can abruptly interrupt a young person’s school-based learning and peer interactions [37], and have an negative impact on their scholastic performance.

The current study demonstrated that as injury severity increased, generally the risk of not achieving the NMS for NAPLAN assessments increased for injured young people compared to their matched peers. Likewise, Azzam et al. [4] demonstrated that increased severity in burn injuries was associated with a decrease in performance on NAPLAN assessments. Sustaining a moderate or severe TBI has previously been found to have consequences for a young person’s academic performance at school [14, 38, 39]. Previous research has also indicated that students who sustained a mild or moderate TBI had fewer cognitive and academic deficits, and a greater recovery, than students with a severe TBI [38].

A TBI can be damaging to language-based cognition [40], as was found in the current study with a higher risk of injured young people not achieving the NMS for two language-associated NAPLAN assessments compared to their matched counterparts. A TBI can have a persistent and chronic impact on a young person’s academic achievements [41], although the academic impact may be of shorter duration for less severe injuries [42].

Recovery from injury can be unpredictable. Some young people may fully recover from their injury, while for others recovery may not take a steady course [43]. After some initial improvement, recovery may plateau [41] or change over time [36]. It is possible that targeted educational support during the post-injury recovery period may mitigate any potential for negative effects on academic performance, particularly for young people with a serious injury who may have higher support needs [44]. For some young people there may be an increasing need for targeted supportive learning services (e.g. tutoring, peer-support shared learning), with increasing time since injury [45]. It will then be important to be able to identify young people at-risk of having difficulties at school due to their injury, so they can be supported. Screening and assessment of injured young people, particularly those who sustained serious injuries, to identify those who will most likely need academic support may be beneficial, along with monitoring their return-to-school progress and performance post-injury to identify ongoing learning support requirements and unmet needs [5, 45, 46]. For example, Kingery et al. [13] demonstrated that 69% of young people with a TBI still had education support needs 7 years after their injury. Return-to-school protocols may provide a guide to a progressive staged approach to return to school post-injury [43, 47] for young people who sustained a serious injury.

The risk of not completing high school was found to be almost twice as high for injured young people compared to their matched peers, potentially indicating the impact of injury early in life may have long-term effects on academic achievement. Not completing high school can have ramifications in later life, affecting the range of employment opportunities, and overall quality of life [44]. Previous research found that young people with a TBI were up to three times less likely to complete high school than national norms [48], and that young people with severe TBI were more likely to need educational support, be less likely to be working in a skilled professional role, and had reduced quality of life in later life [49].

Future research in this area could consider examining group-based trajectories of scholastic performance over time by injury type and/or injury severity to gain an understanding of the impact of different types of injuries at different developmental stages. The utilisation of health services post-injury could also be examined by injury type and severity to identify ongoing health service needs. Other potential explanatory factors could also be considered including family functioning, peer support, time since injury event, time absent from school, and motivation and engagement with school in the post-injury phase. An examination of time since injury event could attempt to distinguish the characteristics associated with young people who may be at an increasing need for supportive learning services with increasing time since injury. Interviews with injured students and their families regarding the impact of the injury on their school and personal life could also be considered.

While the strengths of this study include that it was a large population-based study that covered a 13 year period, there were some limitations. There is the potential that a hospital admission in itself could have an adverse impact on academic performance. For this reason, a secondary analysis was conducted using the existing data by splitting the comparison group into (i) young people who had a non-injury hospitalisation and (ii) young people who had not been hospitalised at all during the study time period, and comparing the academic performance of each group to their matched injured cases. Compared to the matched comparison group that had been hospitalised, the risk of not achieving the NMS remained higher for young people that had been hospitalised for an injury. Although, the risk of not achieving the NMS was highest for injured young people where their matched counterpart had not been hospitalised (Supplementary Fig. 3).

A small proportion of residential postcodes were not known and socioeconomic status was not able to be identified for these young people. While postcode of residence for some young people was used as a matching variable, the CHeReL obtained some residential postcodes from other datasets available in their Master Linkage Key that were not available to the authors. In identifying the matched comparison cohort, the recency of postcode of usual residence may have varied between data collections. For example, postcode of residence at birth could vary from postcode of residence while at school. Only health conditions that are relevant to a hospital admission are indicated in hospital diagnosis classifications, so it is possible that some conditions experienced by young people could be under-enumerated. In addition, around 50% in the non-injured comparison cohort had not been admitted to hospital during the study timeframe, so there was no opportunity to identify comorbid conditions, despite using a 3-year lookback period. This study only included young people who had been hospitalised for an injury, so did not include young people presenting solely to other medical professionals, such as general practitioners, for treatment. However, young people who are hospitalised for their injury are likely to be the most seriously affected. Compared to minor injuries, there were low numbers of young people who sustained moderate or serious injuries, with wide confidence intervals around the relative risks for these injuries that should be interpreted with caution.

No data validity assessments were able to be conducted and it is possible that there could be some data misclassification. The ED visit data did not contain information on ED visits to private hospitals. However, almost all (93%) of ED services are provided by public hospitals in Australia [50]. Pre-injury cognitive performance, physiological measures (such as Glasgow Coma Scale), and measures of family functioning were not available. A higher proportion of injured young people were absent for a NAPLAN assessment compared to their matched peers and higher proportion of injured young people were absent for the assessment over time. The current study could not take into account school absences or school clustering with 1 to 248 young people attending each school. There is potential for students to perform better on the NAPLAN Year 9 writing assessment in 2018 for online assessments as the students were able to revise and edit their task. No information was available regarding any special education services or tutoring received by a young person. There is also potential for unmeasured confounding to influence academic performance.

## Conclusion

Injured young people are demonstrating poorer performance on school assessments, with increasing injury severity having a greater negative impact, compared to their matched counterparts. Injured young people also had almost twice the risk of not completing high school compared to their matched peers. The identification of characteristics of young people most likely to encounter problems in the academic environment after sustaining an injury is important to facilitate the identification of learning support needs. Assessing learning needs and monitoring return-to-school progress post-injury may aid identification of any ongoing support requirements and unmet needs.

## Supplementary Information

**Additional file 1.**

## Data Availability

The data that support the findings of this study are available from the NSW Health Department, NSW Department of Education and NSW Education Standards Authority. Restrictions apply to the availability of these data, which were used under licence for the current study, so are not publicly available.
