# Supplementary material for: The impact of childhood injury and injury severity on school performance and high school completion in Australia: a matched population-based retrospective cohort study
Source: BMC Pediatr. 2021 Sep 25;21:426. doi: 10.1186/s12887-021-02891-x (PMC8464154; doi:10.1186/s12887-021-02891-x)
Supplement: Supplementary file 1 — Additional file 1. [file 12887_2021_2891_MOESM1_ESM.zip › Supplementary data.docx]

**Supplementary Figure 1: Flow chart of study population for NAPLAN assessments**

**Supplementary Figure 2: Flow chart of study population for high school completions**

**Supplementary Figure 3: Multilevel model of characteristics associated with a below NMS NAPLAN assessment for young people with an index injury hospitalisations during 2005-2018 compared to a matched comparison by hospitalisation status and assessment type, linked health and school performance data NSW**^1-3^

Insert Figure 3

^1^Adjusted for injury status, NAPLAN grade, school sector, sex, health condition, LBOTE, socioeconomic status, parental education, and hospital LOS. Numeracy, reading, spelling, grammar and writing type III tests of fixed effects for injury status p<0.0001. Adjusted relative risk excludes 257 with missing LBOTE and 24 home schooled. Writing excludes comorbidity status due to low cell size.

^2^Adjusted for injury status, NAPLAN grade, school sector, sex, health condition, LBOTE, socioeconomic status, and parental education. Numeracy, reading, spelling, grammar and writing type III tests of fixed effects for injury status p<0.0001. Adjusted relative risk excludes 88 with missing socioeconomic status, 175 with missing LBOTE and 27 home schooled. Writing excludes comorbidity status due to low cell size.

^3^The non-injured comparison group was divided into two groups: those who had been hospitalised for a non-injury (n=39,751) and those that had not been hospitalised (n=34,598) during the study timeframe. The GLIMM analysis was then performed for each comparison group separately and their NAPLAN assessment performance was compared to their matched injured case.

**Supplementary Table 1: Number of young people absent or withdrawn from a NAPLAN assessment by grade, linked health and school performance data NSW, 2005-2018**

|  | **Grade 3** | | | | **Grade 5** | | | | **Grade 7** | | | | **Grade 9** | | | |
| --- | --- | --- | --- | --- | --- | --- | --- | --- | --- | --- | --- | --- | --- | --- | --- | --- |
|  | **Injury case** | | **Comparison** | | **Injury case** | | **Comparison** | | **Injury case** | | **Comparison** | | **Injury case** | | **Comparison** | |
|  | **n** | **%**^1^ | **n** | **%**^1^ | **n** | **%**^1^ | **n** | **%**^1^ | **n** | **%**^1^ | **n** | **%**^1^ | **n** | **%**^1^ | **n** | **%**^1^ |
| **Absent for NAPLAN assessment** | |  |  |  |  |  |  |  |  |  |  |  |  |  |  |  |
| Numeracy | 2,772 | 2.3 | 2,060 | 1.9 | 2,439 | 2.4 | 1,841 | 2.0 | 3,349 | 4.2 | 2,210 | 3.2 | 4,365 | 7.7 | 2,530 | 5.3 |
| Reading | 2,319 | 1.9 | 1,635 | 1.5 | 1,955 | 1.9 | 1,463 | 1.6 | 2,778 | 3.5 | 1,790 | 2.6 | 3,767 | 6.7 | 2,157 | 4.5 |
| Spelling and grammar | 2,160 | 1.8 | 1,495 | 1.4 | 1,854 | 1.8 | 1,330 | 1.5 | 2,530 | 3.2 | 1,619 | 2.3 | 3,551 | 6.3 | 1,977 | 4.2 |
| Writing (2011-2018) | 1,861 | 2.2 | 1,309 | 1.7 | 1,834 | 2.1 | 1,302 | 1.6 | 2,690 | 3.4 | 1,668 | 2.4 | 3,636 | 6.4 | 2,060 | 4.3 |
|  |  |  |  |  |  |  |  |  |  |  |  |  |  |  |  |  |
| **Withdrawn from NAPLAN assessment** | | |  |  |  |  |  |  |  |  |  |  |  |  |  |  |
| Numeracy | 1,382 | 1.2 | 960 | 0.9 | 808 | 0.8 | 612 | 0.7 | 506 | 0.6 | 376 | 0.5 | 603 | 1.1 | 403 | 0.9 |
| Reading | 1,463 | 1.2 | 1,038 | 1.0 | 842 | 0.8 | 637 | 0.7 | 516 | 0.7 | 371 | 0.5 | 584 | 1.0 | 400 | 0.8 |
| Spelling and grammar | 1,438 | 1.2 | 1,025 | 1.0 | 848 | 0.8 | 636 | 0.7 | 511 | 0.6 | 361 | 0.5 | 564 | 1.0 | 386 | 0.8 |
| Writing (2011-2018) | 1,233 | 1.5 | 895 | 1.1 | 852 | 1.0 | 633 | 0.8 | 525 | 0.7 | 366 | 0.5 | 574 | 1.0 | 390 | 0.8 |

^1^ Percent calculated for young people in injury and comparison cohorts available to take each NAPLAN assessment.

**Supplementary Table 2: Health conditions and ICD-10-AM classifications**

| **Health condition** | **ICD-10-AM classifications** |
| --- | --- |
| **Circulatory system** |  |
| Hypertension | I10-I15 |
| **Digestive system and allergies** |  |
| Celiac disease and other serious allergies | K52.2, K90.0, T78.0, T78.2, T78.4 |
| **Endocrine, nutritional and metabolic conditions** |  |
| Diabetes | E09-E14 |
| Obesity | E66 |
| Cystic fibrosis | E84 |
| **Immune system conditions and coagulation defects** |  |
| Anaemia | D50-D53 and D55-D64 |
| Coagulation defects (e.g. haemophilia) | D65-C68 |
| **Mental health conditions** |  |
| Autism spectrum disorders | F84 |
| Behavioural and emotional disorders of childhood | F90-F98 |
| Cognitive and behavioural delay | F80-F83 and F88-F89 |
| Eating disorders | F50 |
| Hyperkinetic disorder | F90 |
| Mental retardation | F70-F79 |
| Mood affective disorders | F30-F39 |
| Neurotic, stress-related and somatoform disorders | F40-F48 |
| Personality disorders | F60-F69 |
| Schizophrenia, schizotypal and delusions disorders | F20-F29 |
| **Neoplasms** |  |
| All malignancies | C00-D48 |
| *Acute lymphoblastic leukaemia and acute myeloid leukaemia* | C91.0, C92.0 |
| *Brain cancer* | C71 |
| **Nervous system conditions** |  |
| Cerebral palsy | G80 |
| Epilepsy | G40 |
| **Renal conditions** | I12.0, I13.1, NO3, NO5, N18-N19, N25.0, Z49, Z94.0, Z99.2 |
| **Respiratory conditions** |  |
| Chronic lower respiratory disease | J40-J47 |
| *Asthma* | J45 |

**Supplementary Table 3: Adjusted relative risk of a below NMS NAPLAN assessment for injured young people compared to a matched comparison for grades 3 and 5 by assessment, linked health and school performance data NSW, 2005-2018**

|  |  | **Numeracy** | | **Reading** | | **Spelling** | | **Grammar** | | **Writing (Persuasive 2011-2018)** | |
| --- | --- | --- | --- | --- | --- | --- | --- | --- | --- | --- | --- |
|  | **n**^1^ | **ARR**^2^ | **95%CI** | **ARR**^2^ | **95%CI** | **ARR**^2^ | **95%CI** | **ARR**^2^ | **95%CI** | **ARR**^2^ | **95%CI** |
| **Grade 3** |  |  |  |  |  |  |  |  |  |  |  |
| **Injury**^3^ | 49,934 | 1.19 | 1.12-1.27 | 1.24 | 1.17-1.31 | 1.23 | 1.16-1.30 | 1.27 | 1.20-1.34 | 1.27 | 1.18-1.37 |
| **Injury severity** |  |  |  |  |  |  |  |  |  |  |  |
| Mild^4^ | 47,311 | 1.18 | 1.10-1.26 | 1.22 | 1.15-1.30 | 1.22 | 1.14-1.29 | 1.26 | 1.19-1.33 | 1.26 | 1.16-1.37 |
| Moderate^5^ | 1,914 | 1.24 | 0.94-1.64 | 1.39 | 1.08-1.80 | 1.36 | 1.05-1.78 | 1.19 | 0.93-1.53 | 1.13 | 0.81-1.58 |
| Serious^6^ | 709 | 2.10 | 1.26-3.49 | 1.53 | 0.98-2.40 | 2.13 | 1.34-3.39 | 1.97 | 1.32-2.94 | 2.61 | 1.45-4.67 |
| **TBI**^6,7^ | 1,328 | 1.58 | 1.10-2.26 | 1.41 | 1.01-1.95 | 1.68 | 1.20-2.35 | 1.57 | 1.14-2.17 | 1.78 | 1.19-2.66 |
| **Grade 5** |  |  | |  | |  | |  | |  | |
| **Injury** | 45,899 | 1.30 | 1.22-1.39 | 1.21 | 1.14-1.28 | 1.23 | 1.17-1.31 | 1.23 | 1.16-1.30 | 1.22 | 1.16-1.28 |
| **Injury severity** |  |  |  |  |  |  |  |  |  |  |  |
| Mild | 43,665 | 1.28 | 1.20-1.37 | 1.19 | 1.12-1.26 | 1.22 | 1.15-1.29 | 1.21 | 1.14-1.28 | 1.21 | 1.15-1.28 |
| Moderate | 1,663 | 1.30 | 0.96-1.76 | 1.29 | 1.01-1.65 | 1.42 | 1.09-1.84 | 1.52 | 1.17-1.98 | 1.28 | 1.02-1.62 |
| Serious^6^ | 571 | 3.09 | 1.67-5.69 | 2.43 | 1.54-3.85 | 1.66 | 1.04-2.65 | 1.59 | 1.04-2.42 | 1.93 | 1.25-2.99 |
| **TBI**^6,7^ | 1,262 | 1.63 | 1.11-2.39 | 1.76 | 1.26-2.47 | 1.44 | 1.03-1.99 | 1.67 | 1.21-2.29 | 1.16 | 0.88-1.54 |

^1^ n=number of injured and excludes young people with missing values for socioeconomic status and LBOTE. ^2^ Adjusted for injury status, sex, comorbidity status, LBOTE, socioeconomic status, parental education and hospital LOS, with reference groups of not injured, male, no comorbidities, English-speaking background, least disadvantaged, and bachelor or higher degree, respectively. ^3^ Spelling and writing exclude comorbidity status. ^4^ Writing excludes comorbidity status. ^5^ Reading, spelling and writing exclude comorbidity status. ^6^ Excludes comorbidity status. ^7^ Traumatic brain injury.

**Supplementary Table 4: Adjusted relative risk of a below NMS NAPLAN assessment for injured young people compared to a matched comparison for grades 7 and 9 by assessment, linked health and school performance data NSW, 2005-2018**

|  |  | **Numeracy** | | **Reading** | | **Spelling** | | **Grammar** | | **Writing (Persuasive 2011-2018)** | |
| --- | --- | --- | --- | --- | --- | --- | --- | --- | --- | --- | --- |
|  | **n**^1^ | **ARR**^2^ | **95%CI** | **ARR**^2^ | **95%CI** | **ARR**^2^ | **95%CI** | **ARR**^2^ | **95%CI** | **ARR**^2^ | **95%CI** |
| **Grade 7** |  |  |  |  |  |  |  |  |  |  |  |
| **Injury** | 36,899 | 1.31 | 1.19-1.43 | 1.11 | 1.03-1.19 | 1.17 | 1.10-1.25 | 1.18 | 1.12-1.25 | 1.15 | 1.09-1.20 |
| **Injury severity** |  |  |  |  |  |  |  |  |  |  |  |
| Mild | 35,139 | 1.30 | 1.18-1.43 | 1.09 | 1.02-1.18 | 1.16 | 1.09-1.24 | 1.17 | 1.10-1.24 | 1.13 | 1.08-1.19 |
| Moderate^3^ | 1,331 | 1.28 | 0.86-1.92 | 1.32 | 0.97-1.80 | 1.43 | 1.07-1.91 | 1.35 | 1.04-1.74 | 1.40 | 1.12-1.76 |
| Serious | 429 | 2.00 | 0.96-4.16 | 1.38 | 0.87-2.19 | 1.17 | 0.70-1.94 | 1.42 | 0.92-2.20 | 1.35 | 0.93-1.98 |
| **TBI**^4^ | 1,116 | 1.03 | 0.63-1.70 | 1.42 | 0.94-2.14 | 1.32 | 0.93-1.86 | 1.37 | 0.98-1.92 | 1.21 | 0.93-1.56 |
| **Grade 9** |  |  | |  | |  | |  | |  | |
| **Injury** | 24,458 | 1.17 | 1.03-1.34 | 1.14 | 1.05-1.24 | 1.14 | 1.06-1.23 | 1.11 | 1.04-1.19 | 1.10 | 1.05-1.16 |
| **Injury severity** |  |  |  |  |  |  |  |  |  |  |  |
| Mild^5^ | 23,345 | 1.14 | 0.99-1.30 | 1.09 | 1.00-1.19 | 1.13 | 1.05-1.22 | 1.08 | 1.01-1.16 | 1.08 | 1.03-1.14 |
| Moderate | 859 | 1.60 | 0.86-2.99 | 1.91 | 1.21-3.02 | 1.12 | 0.82-1.53 | 1.27 | 0.92-1.76 | 1.35 | 1.07-1.70 |
| Serious^6^ | 277 | 2.37 | 0.79-7.09 | 2.06 | 0.91-4.71 | 2.15 | 1.10-4.19 | 1.76 | 0.97-3.20 | 1.17 | 0.78-1.74 |
| **TBI**^4,6^ | 806 | 2.21 | 0.94-5.24 | 1.58 | 0.97-2.56 | 1.94 | 1.26-3.00 | 1.44 | 0.98-2.11 | 1.20 | 0.93-1.55 |

^1^ n=number of injured and excludes young people with missing values for socioeconomic status and LBOTE. ^2^ Adjusted for injury status, sex, comorbidity status, LBOTE, socioeconomic status, parental education and hospital LOS with reference groups of not injured, male, no comorbidities, English-speaking background, least disadvantaged, and bachelor or higher degree, respectively. ^3^ Spelling and writing exclude comorbidity status. ^4^ Traumatic brain injury. ^5^Reading, grammar and writing exclude LBOTE and comorbidity status. ^6^Excludes LBOTE and comorbidity status.

**Supplementary Table 5: Multilevel model of characteristics associated with a below NMS NAPLAN assessment for young people with an index minor injury hospitalisation during 2005-2018 compared to a matched comparison by assessment, linked health and school performance data NSW**

|  | **Numeracy**^1^ | | **Reading**^2^ | | **Spelling**^1^ | | **Grammar**^1^ | | **Writing (Persuasive 2011-2018)** ^3^ | |
| --- | --- | --- | --- | --- | --- | --- | --- | --- | --- | --- |
|  | **ARR**^4^ | **95%CI** | **ARR**^4^ | **95%CI** | **ARR**^4^ | **95%CI** | **ARR**^4^ | **95%CI** | **ARR**^4,5^ | **95%CI** |
| **Injury** |  |  |  |  |  |  |  |  |  |  |
| No | 1 |  | 1 |  | 1 |  | 1 |  | 1 |  |
| Yes | 1.11 | 1.06-1.17 | 1.08 | 1.03-1.13 | 1.12 | 1.08-1.17 | 1.10 | 1.05-1.14 | 1.07 | 1.03-1.11 |
| **Gender** |  |  |  |  |  |  |  |  |  |  |
| Male | 1 |  | 1 |  | 1 |  | 1 |  | 1 |  |
| Female | 0.79 | 0.77-0.82 | 0.55 | 0.53-0.57 | 0.48 | 0.47-0.50 | 0.55 | 0.54-0.57 | 0.41 | 0.39-0.42 |
| **Health condition**^2^ |  |  |  |  |  |  |  |  |  |  |
| No | 1 |  | 1 |  | 1 |  | 1 |  |  |  |
| Yes | 1.72 | 1.51-1.95 | 1.60 | 1.43-1.78 | 1.57 | 1.42-1.74 | 1.51 | 1.37-1.67 | - | - |
| **LBOTE** |  |  |  |  |  |  |  |  |  |  |
| No | 1 |  | 1 |  | 1 |  | 1 |  | 1 |  |
| Yes | 0.91 | 0.87-0.95 | 0.82 | 0.79-0.86 | 0.61 | 0.58-0.64 | 0.80 | 0.78-0.83 | 0.63 | 0.61-0.65 |
| **Socioeconomic status** |  |  |  |  |  |  |  |  |  |  |
| Most disadvantaged | 0.31 | 0.29-0.33 | 0.33 | 0.31-0.35 | 0.35 | 0.33-0.37 | 0.37 | 0.35-0.38 | 0.39 | 0.38-0.41 |
| 2 | 0.40 | 0.37-0.43 | 0.42 | 0.39-0.44 | 0.44 | 0.41-0.46 | 0.45 | 0.43-0.47 | 0.46 | 0.44-0.48 |
| 3 | 0.50 | 0.56-0.53 | 0.50 | 0.47-0.53 | 0.53 | 0.50-0.56 | 0.54 | 0.51-0.57 | 0.54 | 0.52-0.57 |
| 4 | 0.62 | 0.57-0.68 | 0.63 | 0.59-0.69 | 0.66 | 0.62-0.71 | 0.66 | 0.62-0.70 | 0.66 | 0.63-0.71 |
| Least disadvantaged | 1 |  | 1 |  | 1 |  | 1 |  | 1 |  |
| **NAPLAN Grade** |  |  |  |  |  |  |  |  |  |  |
| 3 | 1 |  | 1 |  | 1 |  | 1 |  | 1 |  |
| 5 | 1.33 | 1.27-1.39 | 1.19 | 1.15-1.23 | 0.87 | 0.85-0.90 | 0.87 | 0.84-0.89 | 0.72 | 0.70-0.74 |
| 7 | 1.61 | 1.52-1.71 | 0.90 | 0.87-0.93 | 0.66 | 0.64-0.68 | 0.69 | 0.67-0.71 | 0.48 | 0.47-0.50 |
| 9 | 1.12 | 1.08-1.17 | 1.25 | 1.20-1.29 | 1.19 | 1.15-1.23 | 1.21 | 1.17-1.25 | 2.22 | 2.14-2.31 |
| **Parental education** |  |  |  |  |  |  |  |  |  |  |
| Bachelor/higher degree | 1 |  | 1 |  | 1 |  | 1 |  | 1 |  |
| Other | 0.37 | 0.35-0.39 | 0.35 | 0.33-0.36 | 0.40 | 0.39-0.42 | 0.37 | 0.36-0.39 | 0.43 | 0.41-0.44 |
| **School sector** |  |  |  |  |  |  |  |  |  |  |
| Government | 1 |  | 1 |  | 1 |  | 1 |  | 1 |  |
| Catholic | 1.01 | 0.92-0.12 | 0.98 | 0.91-1.06 | 1.00 | 0.94-1.08 | 1.03 | 0.96-1.10 | 0.92 | 0.87-1.00 |
| Independent | 0.41 | 0.39-0.44 | 0.46 | 0.44-0.48 | 0.53 | 0.51-0.55 | 0.51 | 0.49-0.53 | 0.47 | 0.45-0.49 |

^1^Numeracy, spelling, and grammar type III tests of fixed effects: Injury p<0.0001; Gender p<0.0001; Health condition p<0.0001; LBOTE p<0.0001; socioeconomic status p<0.0001; NAPLAN grade p<0.0001; Parental education p<0.0001; School sector p<0.0001; and hospital LOS p<0.0001. ^2^Reading type III tests of fixed effects: Injury p<0.0008; Gender p<0.0001; Health condition p<0.0001; LBOTE p<0.0001; socioeconomic status p<0.0001; NAPLAN grade p<0.0001; Parental education p<0.0001; School sector p<0.0001; and hospital LOS p<0.0001. ^3^Writing type III tests of fixed effects: Injury p<0.0003; Gender p<0.0001; Health condition p<0.0001; LBOTE p<0.0001; socioeconomic status p<0.0001; NAPLAN grade p<0.0001; Parental education p<0.0001; School sector p<0.0001; and hospital LOS p<0.0001. ^4^Adjusted relative risk excludes 174 with missing socioeconomic status, 808 with missing LBOTE and 90 home schooled young people. ^5^Writing excludes comorbidity status due to low cell size.

**Supplementary Table 6: Multilevel model of characteristics associated with a below NMS NAPLAN assessment for young people with an index moderate injury hospitalisation during 2005-2018 compared to a matched comparison by assessment, linked health and school performance data NSW**

|  | **Numeracy**^1^ | | **Reading**^2^ | | **Spelling**^3^ | | **Grammar**^4^ | | **Writing (Persuasive 2011-2018)** ^5^ | |
| --- | --- | --- | --- | --- | --- | --- | --- | --- | --- | --- |
|  | **ARR**^6^ | **95%CI** | **ARR**^6^ | **95%CI** | **ARR**^6,7^ | **95%CI** | **ARR**^6^ | **95%CI** | **ARR**^6,7^ | **95%CI** |
| **Injury** |  |  |  |  |  |  |  |  |  |  |
| No | 1 |  | 1 |  | 1 |  | 1 |  | 1 |  |
| Yes | 1.05 | 0.85-1.29 | 1.22 | 1.01-1.48 | 1.24 | 1.03-1.49 | 1.24 | 1.03-1.48 | 1.24 | 1.06-1.46 |
| **Gender** |  |  |  |  |  |  |  |  |  |  |
| Male | 1 |  | 1 |  | 1 |  | 1 |  | 1 |  |
| Female | 0.72 | 0.60-0.85 | 0.60 | 0.51-0.69 | 0.56 | 0.48-0.65 | 0.65 | 0.56-0.74 | 0.46 | 0.40-0.53 |
| **Health condition**^2^ |  |  |  |  |  |  |  |  |  |  |
| No | 1 |  | 1 |  |  |  | 1 |  |  |  |
| Yes | 1.96 | 1.31-2.93 | 1.61 | 1.12-2.32 | - | - | 1.44 | 1.01-2.06 | - | - |
| **LBOTE** |  |  |  |  |  |  |  |  |  |  |
| No | 1 |  | 1 |  | 1 |  | 1 |  |  |  |
| Yes | 0.99 | 0.82-1.21 | 0.96 | 0.81-1.12 | 0.67 | 0.56-0.81 | 0.80 | 0.68-0.94 | - | - |
| **Socioeconomic status** |  |  |  |  |  |  |  |  |  |  |
| Most disadvantaged | 0.39 | 0.27-0.55 | 0.38 | 0.29-0.51 | 0.51 | 0.40-0.65 | 0.43 | 0.33-0.56 | 0.45 | 0.36-0.56 |
| 2 | 0.49 | 0.34-0.70 | 0.49 | 0.36-0.65 | 0.63 | 0.49-0.81 | 0.54 | 0.41-0.70 | 0.52 | 0.42-0.65 |
| 3 | 0.48 | 0.33-0.68 | 0.50 | 0.37-0.68 | 0.69 | 0.53-0.89 | 0.55 | 0.42-0.72 | 0.63 | 0.50-0.79 |
| 4 | 0.60 | 0.39-0.91 | 0.77 | 0.53-1.12 | 0.92 | 0.66-1.27 | 0.76 | 0.55-1.05 | 0.86 | 0.64-1.15 |
| Least disadvantaged | 1 |  | 1 |  | 1 |  | 1 |  | 1 |  |
| **NAPLAN Grade** |  |  |  |  |  |  |  |  |  |  |
| 3 | 1 |  | 1 |  | 1 |  | 1 |  | 1 |  |
| 5 | 1.34 | 1.10-1.63 | 1.09 | 0.94-1.26 | 0.94 | 0.82-1.07 | 0.82 | 0.72-0.94 | 0.77 | 0.69-0.86 |
| 7 | 1.48 | 1.15-1.91 | 0.97 | 0.81-1.15 | 0.74 | 0.64-0.85 | 0.70 | 0.60-0.82 | 0.53 | 0.47-0.59 |
| 9 | 1.06 | 0.89-1.26 | 1.21 | 1.05-1.41 | 1.26 | 1.09-1.45 | 1.16 | 1.00-1.34 | 2.25 | 1.91-2.65 |
| **Parental education** |  |  |  |  |  |  |  |  |  |  |
| Bachelor/higher degree | 1 |  | 1 |  | 1 |  | 1 |  | 1 |  |
| Other | 0.33 | 0.25-0.43 | 0.33 | 0.26-0.41 | 0.31 | 0.25-0.38 | 0.36 | 0.39-0.44 | 0.32 | 0.27-0.39 |
| **School sector** |  |  |  |  |  |  |  |  |  |  |
| Government | 1 |  | 1 |  | 1 |  | 1 |  | - | - |
| Catholic | 0.98 | 0.62-1.55 | 0.88 | 0.62-1.24 | 1.05 | 0.78-1.40 | 0.98 | 0.72-1.33 | - | - |
| Independent | 0.40 | 0.31-0.52 | 0.44 | 0.35-0.54 | 0.63 | 0.53-0.75 | 0.50 | 0.41-0.60 | - | - |

^1^Numeracy type III tests of fixed effects: Injury p=0.7; Gender p<0.0001; Health condition p<0.001; LBOTE p=0.9; socioeconomic status p<0.0001; NAPLAN grade p<0.002; Parental education p<0.0001; School sector p<0.0001; and hospital LOS p<0.0001. ^2^Reading type III tests of fixed effects: Injury p<0.04; Gender p<0.0001; Health condition p<0.009; LBOTE p=0.6; socioeconomic status p<0.0001; NAPLAN grade p<0.02; Parental education p<0.0001; School sector p<0.0001; and hospital LOS p<0.006. ^3^Spelling type III tests of fixed effects: Injury p<0.02; Gender p<0.0001; LBOTE p<0.0001; socioeconomic status p<0.0001; NAPLAN grade p<0.0001; Parental education p<0.0001; School sector p<0.0001; and hospital LOS p<0.02. ^4^Grammar type III tests of fixed effects: Injury p<0.02; Gender p<0.0001; LBOTE p<0.006; socioeconomic status p<0.0001; NAPLAN grade p<0.0001; Parental education p<0.0001; School sector p<0.0001; and hospital LOS p<0.05. ^5^Writing type III tests of fixed effects: Injury p<0.008; Gender p<0.0001; socioeconomic status p<0.0001; NAPLAN grade p<0.0001; Parental education p<0.0001; School sector p<0.0001; and hospital LOS p<0.0001. ^6^Adjusted relative risk excludes 2 with missing socioeconomic status, 43 with missing LBOTE and 2 home schooled young people. ^7^Spelling excludes comorbidity status and Writing excludes comorbidity status and LBOTE due to small cell sizes.

**Supplementary Table 7: Multilevel model of characteristics associated with a below NMS NAPLAN assessment for young people with an index serious injury hospitalisation during 2005-2018 compared to a matched comparison by assessment, linked health and school performance data NSW**

|  | **Numeracy**^1^ | | **Reading**^2^ | | **Spelling**^3^ | | **Grammar**^4^ | | **Writing (Persuasive 2011-2018)** ^5^ | |
| --- | --- | --- | --- | --- | --- | --- | --- | --- | --- | --- |
|  | **ARR**^6^ | **95%CI** | **ARR**^6^ | **95%CI** | **ARR**^6^ | **95%CI** | **ARR**^6,7^ | **95%CI** | **ARR**^6,7^ | **95%CI** |
| **Injury** |  |  |  |  |  |  |  |  |  |  |
| No | 1 |  | 1 |  | 1 |  | 1 |  | 1 |  |
| Yes | 1.75 | 1.18-2.61 | 1.43 | 1.05-1.96 | 1.42 | 1.02-1.97 | 1.33 | 1.00-1.77 | 1.16 | 0.89-1.51 |
| **Gender** |  |  |  |  |  |  |  |  |  |  |
| Male | 1 |  | 1 |  | 1 |  | 1 |  | 1 |  |
| Female | 1.31 | 1.02-1.67 | 0.67 | 0.52-0.84 | 0.76 | 0.60-0.97 | 0.72 | 0.58-0.89 | 0.59 | 0.48-0.74 |
| **Health condition**^2^ |  |  |  |  |  |  |  |  |  |  |
| No | 1 |  | 1 |  | 1 |  |  |  |  |  |
| Yes | 1.99 | 1.28-3.10 | 1.23 | 0.76-2.00 | 1.35 | 0.82-2.20 | - | - | - | - |
| **LBOTE** |  |  |  |  |  |  |  |  |  |  |
| No | 1 |  | 1 |  | 1 |  | 1 |  |  |  |
| Yes | 0.88 | 0.66-1.18 | 0.83 | 0.64-1.07 | 0.60 | 0.45-0.81 | 0.84 | 0.66-1.07 | - | - |
| **Socioeconomic status** |  |  |  |  |  |  |  |  |  |  |
| Most disadvantaged | 0.39 | 0.24-0.63 | 0.38 | 0.25-0.59 | 0.43 | 0.29-0.65 | 0.38 | 0.25-0.57 | 0.47 | 0.33-0.68 |
| 2 | 0.49 | 0.31-0.80 | 0.43 | 0.28-0.65 | 0.50 | 0.33-0.75 | 0.40 | 0.27-0.61 | 0.47 | 0.33-0.68 |
| 3 | 0.85 | 0.49-1.46 | 0.67 | 0.42-1.08 | 0.67 | 0.43-1.04 | 0.58 | 0.37-0.90 | 0.66 | 0.44-0.98 |
| 4 | 0.90 | 0.46-1.74 | 0.46 | 0.28-0.77 | 0.86 | 0.49-1.50 | 0.53 | 0.32-0.86 | 0.54 | 0.35-0.83 |
| Least disadvantaged | 1 |  | 1 |  | 1 |  | 1 |  | 1 |  |
| **NAPLAN Grade** |  |  |  |  |  |  |  |  |  |  |
| 3 | 1 |  | 1 |  | 1 |  | 1 |  | 1 |  |
| 5 | 1.14 | 0.86-1.52 | 1.00 | 0.81-1.25 | 0.99 | 0.79-1.25 | 0.80 | 0.65-0.99 | 0.66 | 0.55-0.80 |
| 7 | 1.28 | 0.87-1.88 | 0.99 | 0.76-1.31 | 0.68 | 0.53-0.87 | 0.62 | 0.49-0.79 | 0.48 | 0.39-0.59 |
| 9 | 1.11 | 0.30-0.62 | 1.48 | 1.17-1.86 | 1.22 | 0.97-1.54 | 1.06 | 0.85-1.33 | 1.87 | 1.43-2.44 |
| **Parental education** |  |  |  |  |  |  |  |  |  |  |
| Bachelor/higher degree | 1 |  | 1 |  | 1 |  | 1 |  | 1 |  |
| Other | 0.43 | 0.34-0.73 | 0.44 | 0.32-0.59 | 0.40 | 0.29-0.55 | 0.41 | 0.30-0.55 | 0.38 | 0.29-0.50 |
| **School sector** |  |  |  |  |  |  |  |  |  |  |
| Government | 1 |  | 1 |  | 1 |  | 1 |  | - | - |
| Catholic | 1.37 | 0.65-2.87 | 0.96 | 0.54-1.71 | 0.85 | 0.50-1.44 | 1.24 | 0.69-2.24 | - | - |
| Independent | 0.49 | 0.34-0.73 | 0.44 | 0.31-0.61 | 0.47 | 0.34-0.65 | 0.43 | 0.31-0.58 | - | - |

^1^Numeracy type III tests of fixed effects: Injury p<0.006; Gender p<0.03; Health condition p<0.002; LBOTE p=0.4; socioeconomic status p<0.0001; NAPLAN grade p=0.6; Parental education p<0.0001; School sector p<0.0001; and hospital LOS p<0.03. ^2^Reading type III tests of fixed effects: Injury p<0.03; Gender p<0.0008; Health condition p=0.4; LBOTE p=0.2; socioeconomic status p<0.0001; NAPLAN grade p<0.003; Parental education p<0.0001; School sector p<0.0001; and hospital LOS p<0.003. ^3^Spelling type III tests of fixed effects: Injury p<0.04; Gender p<0.02; Health condition p=0.2; LBOTE p<0.0007; socioeconomic status p<0.0001; NAPLAN grade p<0.0001; Parental education p<0.0001; School sector p<0.0001; and hospital LOS p<0.09. ^4^Grammar type III tests of fixed effects: Injury p<0.05; Gender p<0.003; LBOTE p=0.1; socioeconomic status p<0.0001; NAPLAN grade p<0.0001; Parental education p<0.0001; School sector p<0.0001; and hospital LOS p<0.001. ^5^Writing type III tests of fixed effects: Injury p=0.3; Gender p<0.0001; socioeconomic status p<0.0002; NAPLAN grade p<0.0001; Parental education p<0.0001; and hospital LOS p<0.002. ^6^Adjusted relative risk excludes 7 with missing LBOTE. ^7^Grammar excludes comorbidity status and Writing excludes comorbidity status and LBOTE due to small cell sizes and school sector.

**Supplementary Table 8: Multilevel model of characteristics associated with a below NMS NAPLAN assessment for young people with an index TBI hospitalisation during 2005-2018 compared to a matched comparison by assessment, linked health and school performance data NSW**

|  | **Numeracy**^1^ | | **Reading**^2^ | | **Spelling**^3^ | | **Grammar**^4^ | | **Writing (Persuasive 2011-2018)** ^5^ | |
| --- | --- | --- | --- | --- | --- | --- | --- | --- | --- | --- |
|  | **ARR**^6^ | **95%CI** | **ARR**^6^ | **95%CI** | **ARR**^6^ | **95%CI** | **ARR**^6,7^ | **95%CI** | **ARR**^6,7^ | **95%CI** |
| **Injury** |  |  |  |  |  |  |  |  |  |  |
| No | 1 |  | 1 |  | 1 |  | 1 |  | 1 |  |
| Yes | 1.18 | 0.89-1.56 | 1.25 | 0.98-1.59 | 1.38 | 1.08-1.76 | 1.31 | 1.04-1.65 | 1.07 | 0.88-1.30 |
| **Gender** |  |  |  |  |  |  |  |  |  |  |
| Male | 1 |  | 1 |  | 1 |  | 1 |  | 1 |  |
| Female | 0.89 | 0.73-1.08 | 0.56 | 0.46-0.68 | 0.54 | 0.45-0.65 | 0.56 | 0.48-0.67 | 0.41 | 0.34-0.48 |
| **Health condition**^2^ |  |  |  |  |  |  |  |  |  |  |
| No | 1 |  | 1 |  | 1 |  |  |  |  |  |
| Yes | 3.24 | 2.14-4.90 | 1.94 | 1.25-3.02 | 2.09 | 1.41-3.08 | - | - | - | - |
| **LBOTE** |  |  |  |  |  |  |  |  |  |  |
| No | 1 |  | 1 |  | 1 |  | 1 |  | 1 |  |
| Yes | 1.0 | 0.74-1.25 | 0.84 | 0.66-1.06 | 0.58 | 0.45-0.75 | 0.68 | 0.54-0.87 | 0.50 | 0.39-0.63 |
| **Socioeconomic status** |  |  |  |  |  |  |  |  |  |  |
| Most disadvantaged | 0.35 | 0.24-0.51 | 0.38 | 0.28-0.51 | 0.36 | 0.27-0.48 | 0.50 | 0.38-0.66 | 0.43 | 0.33-0.55 |
| 2 | 0.51 | 0.34-0.74 | 0.50 | 0.36-0.68 | 0.47 | 0.35-0.63 | 0.53 | 0.40-0.69 | 0.46 | 0.36-0.59 |
| 3 | 0.42 | 0.28-0.61 | 0.53 | 0.38-0.73 | 0.58 | 0.43-0.78 | 0.64 | 0.48-0.85 | 0.58 | 0.45-0.75 |
| 4 | 0.82 | 0.48-1.40 | 0.74 | 0.48-1.13 | 0.74 | 0.49-1.10 | 0.76 | 0.53-1.10 | 0.66 | 0.48-0.91 |
| Least disadvantaged | 1 |  | 1 |  | 1 |  | 1 |  | 1 |  |
| **NAPLAN Grade** |  |  |  |  |  |  |  |  |  |  |
| 3 | 1 |  | 1 |  | 1 |  | 1 |  | 1 |  |
| 5 | 1.58 | 1.24-2.03 | 1.19 | 0.99-1.43 | 0.94 | 0.80-1.11 | 0.89 | 0.76-1.04 | 0.69 | 0.60-0.80 |
| 7 | 1.63 | 1.21-2.19 | 0.93 | 0.76-1.13 | 0.70 | 0.59-1.11 | 0.75 | 0.63-0.89 | 0.47 | 0.41-0.55 |
| 9 | 1.09 | 0.88-1.35 | 1.26 | 1.04-1.52 | 1.21 | 1.01-1.46 | 1.23 | 1.03-1.48 | 1.75 | 1.42-2.16 |
| **Parental education** |  |  |  |  |  |  |  |  |  |  |
| Bachelor/higher degree | 1 |  | 1 |  | 1 |  | 1 |  | 1 |  |
| Other | 0.57 | 0.44-0.73 | 0.45 | 0.36-0.57 | 0.47 | 0.38-0.59 | 0.41 | 0.33-0.51 | 0.49 | 0.41-0.59 |
| **School sector** |  |  |  |  |  |  |  |  |  |  |
| Government | 1 |  | 1 |  | 1 |  | 1 |  | 1 |  |
| Catholic | 1.16 | 0.67-2.01 | 0.96 | 0.62-1.47 | 0.78 | 0.57-1.09 | 0.96 | 0.67-1.37 | 0.89 | 0.67-1.20 |
| Independent | 0.41 | 0.30-0.56 | 0.41 | 0.32-0.53 | 0.56 | 0.46-0.69 | 0.50 | 0.41-0.62 | 0.52 | 0.44-0.62 |

^1^Numeracy type III tests of fixed effects: Injury p=0.2; Gender p=0.2; Health condition p<0.0001; LBOTE p=0.8; socioeconomic status p<0.0001; NAPLAN grade p<0.0002; Parental education p<0.0001; School sector p<0.0001; and hospital LOS p<0.03. ^2^Reading type III tests of fixed effects: Injury p<0.07; Gender p<0.0001; Health condition p<0.003; LBOTE p=0.1; socioeconomic status p<0.0001; NAPLAN grade p<0.005; Parental education p<0.0001; School sector p<0.0001; and hospital LOS p<0.01. ^3^Spelling type III tests of fixed effects: Injury p<0.009; Gender p<0.0001; Health condition p<0.0002; LBOTE p<0.0001; socioeconomic status p<0.0001; NAPLAN grade p<0.0001; Parental education p<0.0001; School sector p<0.0001; and hospital LOS p=0.3. ^4^Grammar type III tests of fixed effects: Injury p<0.02; Gender p<0.0001; LBOTE p<0.002; socioeconomic status p<0.0001; NAPLAN grade p<0.0001; Parental education p<0.0001; School sector p<0.0001; and hospital LOS p=0.1. ^5^Writing type III tests of fixed effects: Injury p=0.5; Gender p<0.0001; LBOTE p<0.0001; socioeconomic status p<0.0002; NAPLAN grade p<0.0001; Parental education p<0.0001; and hospital LOS p<0.02. ^6^Adjusted relative risk excludes 10 with missing socioeconomic status, 19 with missing LBOTE and 4 home schooled young people. ^7^Grammar and Writing exclude comorbidity status due to small cell sizes.
