# Supplementary material for: The impact of childhood injury and injury severity on school performance and high school completion in Australia: a matched population-based retrospective cohort study
Source: BMC Pediatr. 2021 Sep 25;21:426. doi: 10.1186/s12887-021-02891-x (PMC8464154; doi:10.1186/s12887-021-02891-x)
Supplement: Supplementary file 1 — Additional file 1. [file 12887_2021_2891_MOESM1_ESM.zip › Supplementary Figure 3.pdf]

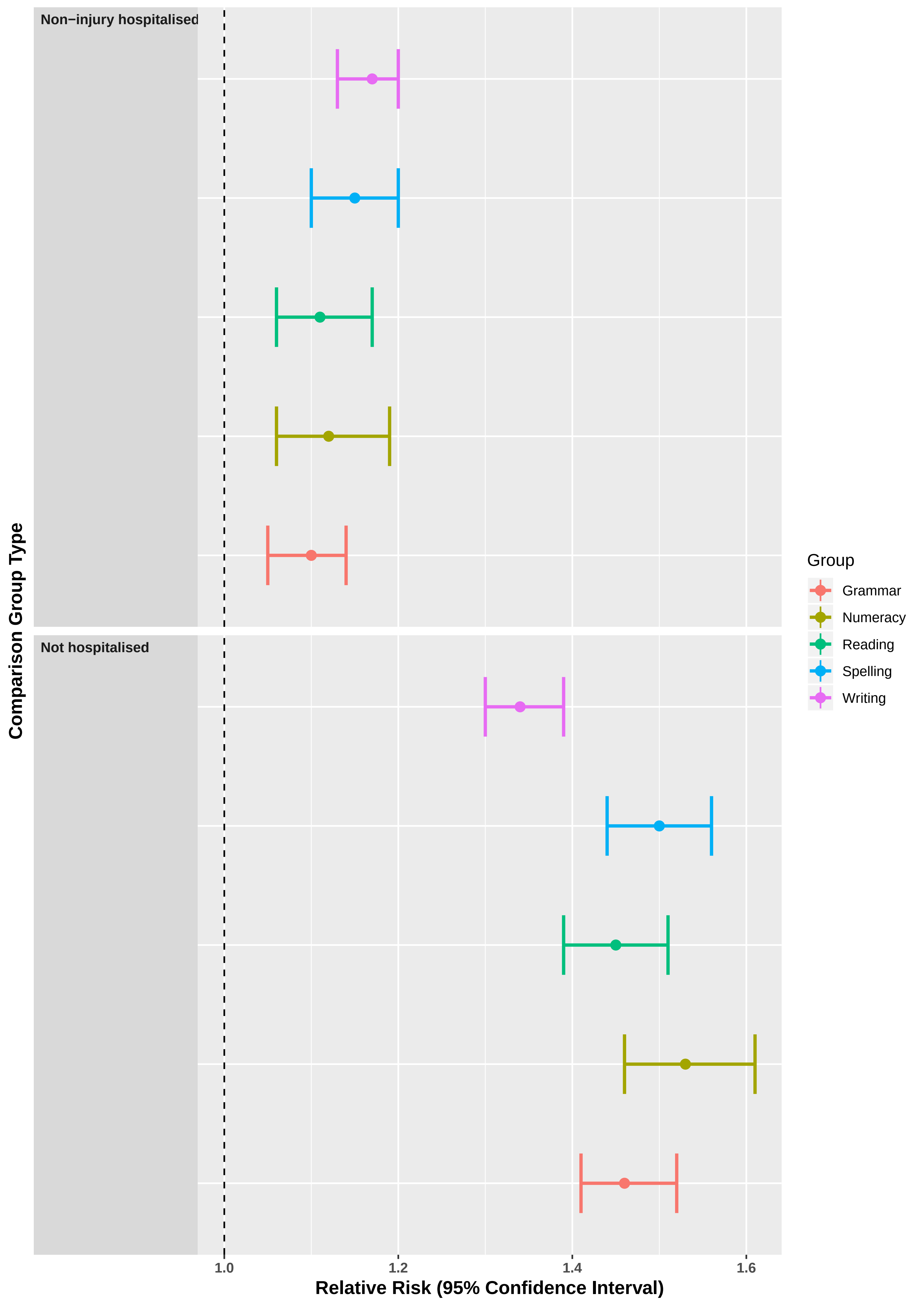

Non−injury hospitalised
Not hospitalised
Comparison Group Type
1.0
1.2
1.4
1.6
Relative Risk (95% Confidence Interval)
Group
Grammar
Numeracy
Reading
Spelling
Writing
