# Supplementary figures and images for: The impact of childhood injury and injury severity on school performance and high school completion in Australia: a matched population-based retrospective cohort study
Source: BMC Pediatr. 2021 Sep 25;21:426. doi: 10.1186/s12887-021-02891-x (PMC8464154; doi:10.1186/s12887-021-02891-x)

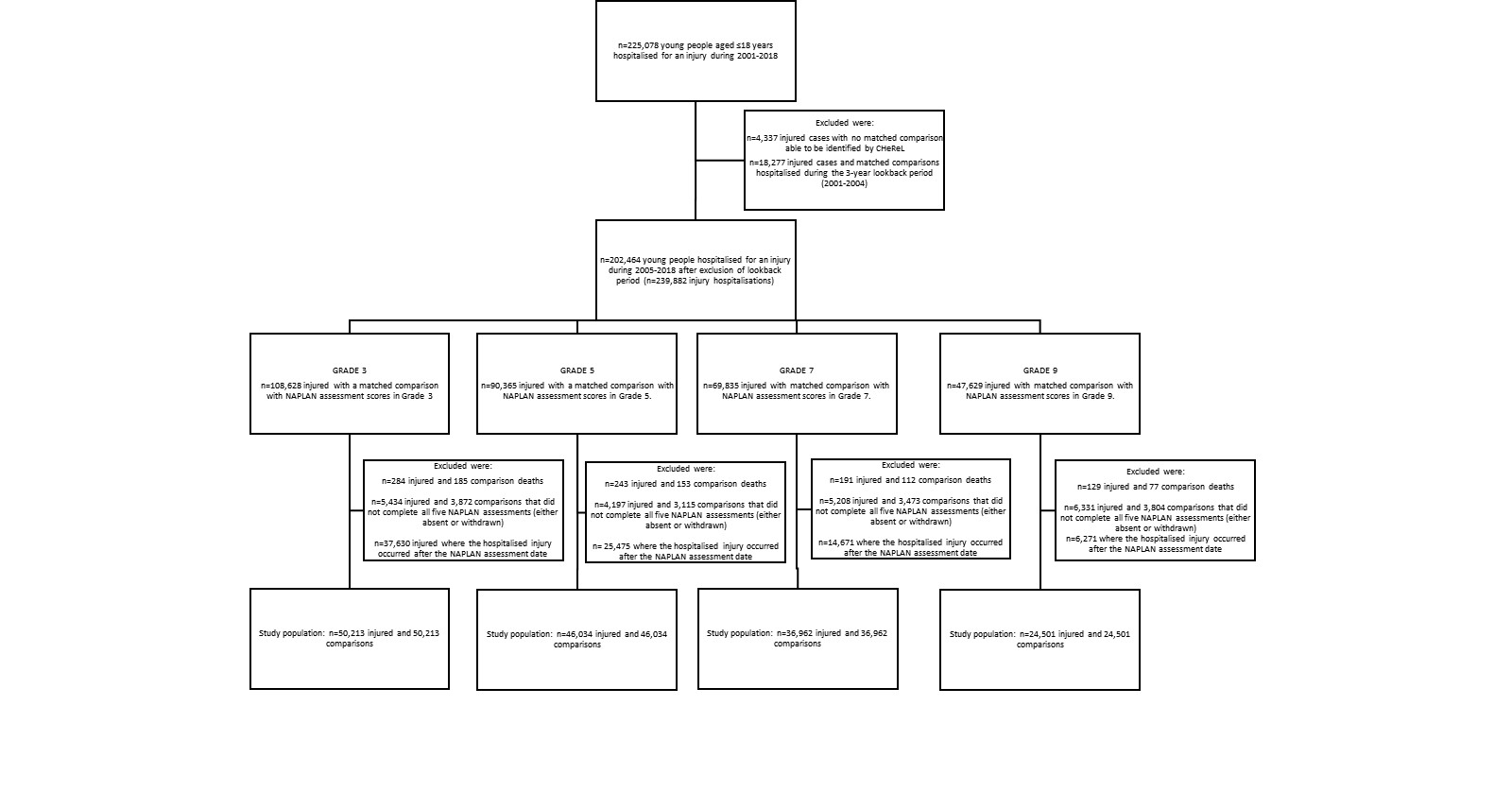

Supplement: Supplementary file 1 — Additional file 1. [file 12887_2021_2891_MOESM1_ESM.zip › Supplementary Figure 1.docx]

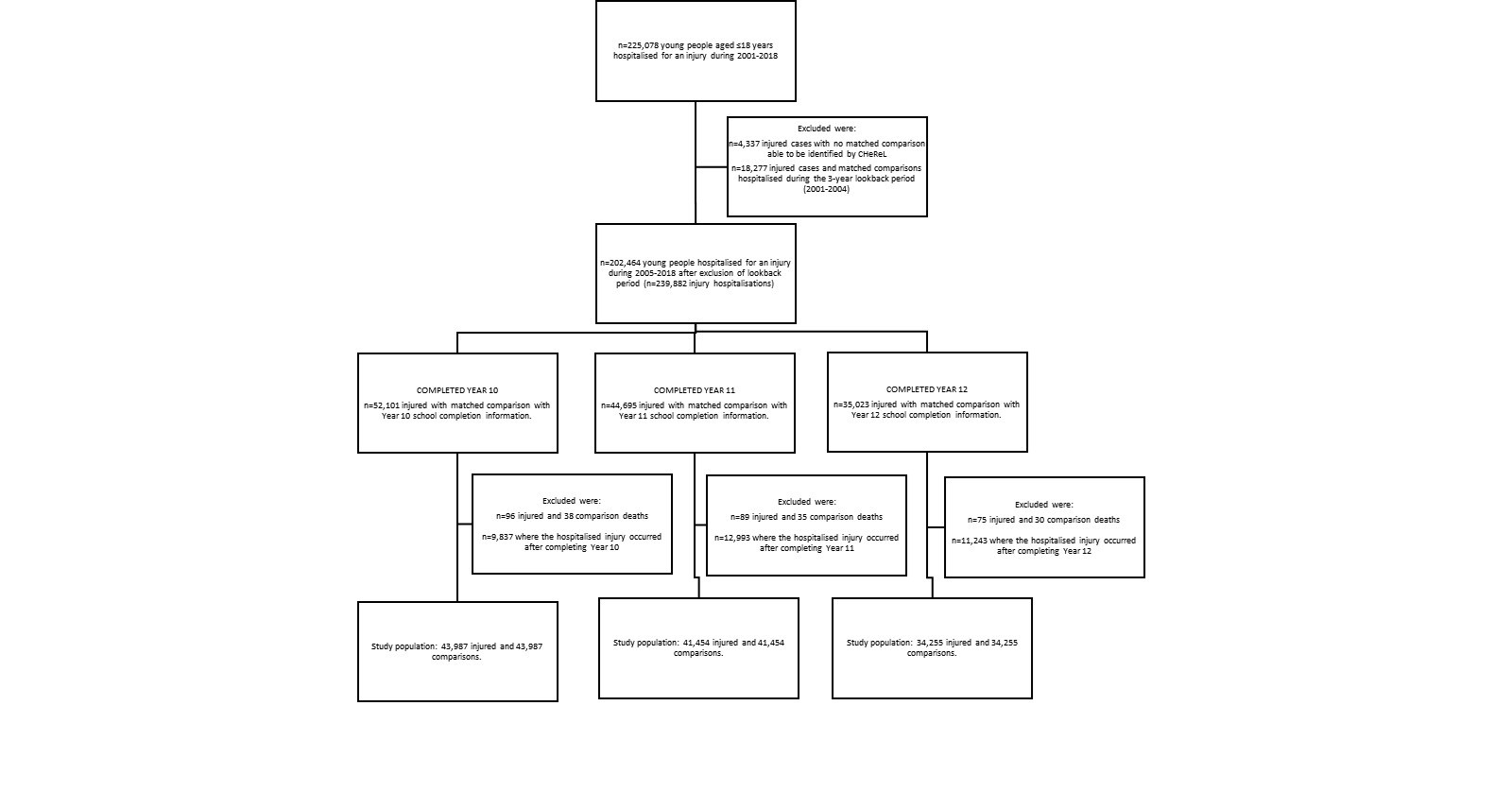

Supplement: Supplementary file 1 — Additional file 1. [file 12887_2021_2891_MOESM1_ESM.zip › Supplementary Figure 2.docx]
